# The role of reciprocal fusions in *MLL*-r acute leukemia: studying the chromosomal translocation t(4;11)

**DOI:** 10.1038/s41388-021-02001-2

**Published:** 2021-09-06

**Authors:** Alexander Wilhelm, Rolf Marschalek

**Affiliations:** Institute of Pharmaceutical Biology/DCAL, Goethe-University of Frankfurt, Frankfurt/Main, Germany

**Keywords:** Cell biology, Genetics

## Abstract

Leukemia patients bearing the t(4;11)(q21;q23) translocations can be divided into two subgroups: those expressing both reciprocal fusion genes, and those that have only the *MLL-AF4* fusion gene. Moreover, a recent study has demonstrated that patients expressing both fusion genes have a better outcome than patients that are expressing the MLL-AF4 fusion protein alone. All this may point to a clonal process where the reciprocal fusion gene *AF4-MLL* could be lost during disease progression, as this loss may select for a more aggressive type of leukemia. Therefore, we were interested in unraveling the decisive role of the AF4-MLL fusion protein at an early timepoint of disease development. We designed an experimental model system where the MLL-AF4 fusion protein was constitutively expressed, while an inducible *AF4-MLL* fusion gene was induced for only 48 h. Subsequently, we investigated genome-wide changes by RNA- and ATAC-Seq experiments at distinct timepoints. These analyses revealed that the expression of AF4-MLL for only 48 h was sufficient to significantly change the genomic landscape (transcription and chromatin) even on a longer time scale. Thus, we have to conclude that the AF4-MLL fusion protein works through a hit-and-run mechanism, probably necessary to set up pre-leukemic conditions, but being dispensable for later disease progression.

## Introduction

*MLL-r* leukemia is diagnosed in 5–10% of all acute leukemia patients, and the spectrum of *MLL* fusion partners has increased over the last 30 years of research to more than 100 [1]. The most frequent translocation in proB ALL is t(4;11)(q21;q23) which represents overall about 57% of all cases. In this particular translocation, the two genes *MLL* (*KMT2A*) and *AF4* (*AFF1*) are fused in a balanced recombination event to cause the generation of the two fusion genes *MLL-AF4* and *AF4-MLL*, respectively. Most of the identified breakpoints of leukemia patients cluster to *MLL* introns 9–11 (~81%), and *AF4* introns 3 and 4 (~86%), indicating that these regions of both genes are the preferred hotspots for the illegitimate recombination event. Of note, breakpoints within *MLL* intron 11 are most frequently found in infant ALL, and appear to change the biology of the reciprocal AF4-MLL fusion protein by disrupting of the first PHD finger of the PHD domain. This changes the binding properties of CYP33 (PPIE, Peptidyl-prolyl cis-trans isomerase E) to the PHD domain, and thus, the biology of the reciprocal fusion protein AF4-MLL [2]. This “infant version” of the AF4-MLL fusion protein has been used throughout this study.

In the past 25 years, researchers have tried to dissect the role of the MLL-AF4 and AF4-MLL fusion proteins. In most studies, only the *MLL-AF4* fusion gene has been tested in functional assays, but most studies have failed to demonstrate the oncogenicity of the MLL-AF4 fusion protein (summarized in ref. [3]). In vivo studies in mice have also mostly failed, except for two studies, one of which comes from our own laboratory. Of note, Lin et al. were only able to recapitulate leukemia development in mice when using a marinized *MLL-Af4* construct in distinct target cells [4]. They also failed with the full-human counterpart to convincingly create leukemia with the *MLL-AF4* fusion gene alone. Our study from 2010 demonstrated that the AF4-MLL fusion protein is indispensable for leukemia onset, as the onset of leukemia was observed only with *AF4-MLL* or both fusion genes, but never with *MLL-AF4* alone [5]. Expression of AF4-MLL alone caused B-/T-type leukemia, but only in one-third of the transplanted mice, which may indicate that other transcription factors (e.g., RUNX1) were somehow complementing functions deriving from the missing *MLL-AF4* allele to drive leukemia [6].

By contrast, when CRISPR/CAS9 technology was used to generate balanced chromosomal translocations in target cells, leukemia development was efficient [7]. This raises again the general questions about the requirements of the direct and reciprocal fusion protein and their roles for leukemia onset (for review see ref. [3]).

In one sense, t(4;11) leukemia is quite a peculiar disease because leukemic cells do not display recurrent secondary mutations [8, 9]—at least in many infant t(4;11) leukemia cases [10]. Apart from a few RAS mutations [11, 12] or individual subclonal mutations, the overall mutation frequency is very low. Thus, this initial translocation event seems to be necessary and sufficient to cause the onset of acute leukemia. However, the question remains what kind of functions are exerted by both fusion proteins and whether their actions are required temporarily or throughout the whole process of leukemia onset.

MLL and AF4 wildtype protein complexes have some very basic functions in mammalian cells. The MLL wildtype protein complex is known to confer active chromatin marks on target gene promotors which enable target gene transcription [13–15]. The AF4 complex [16, 17], also termed “super-elongation complex” (reviewed in ref. [18]), is responsible for transcriptional elongation [19]. Both biological processes are crucial for any living cell, and therefore, pathological functions deriving from t(4;11) fusion proteins should be easily monitored when investigating changes in gene transcription. This is important to mention as we did not aim to mimic leukemia development, rather study the immediate changes in chromatin and gene transcription in combination with long-time effects.

Moreover, we were also interested in finding a rational explanation for the elimination of the *AF4-MLL* allele in about 40% of all patients that appears to worsen the disease outcome, and what triggers this clonal evolutionary process [20].

## Results

### Cloning of t(4;11) fusion genes and establishment of a t(4;11) model system

**Fig. 1:**
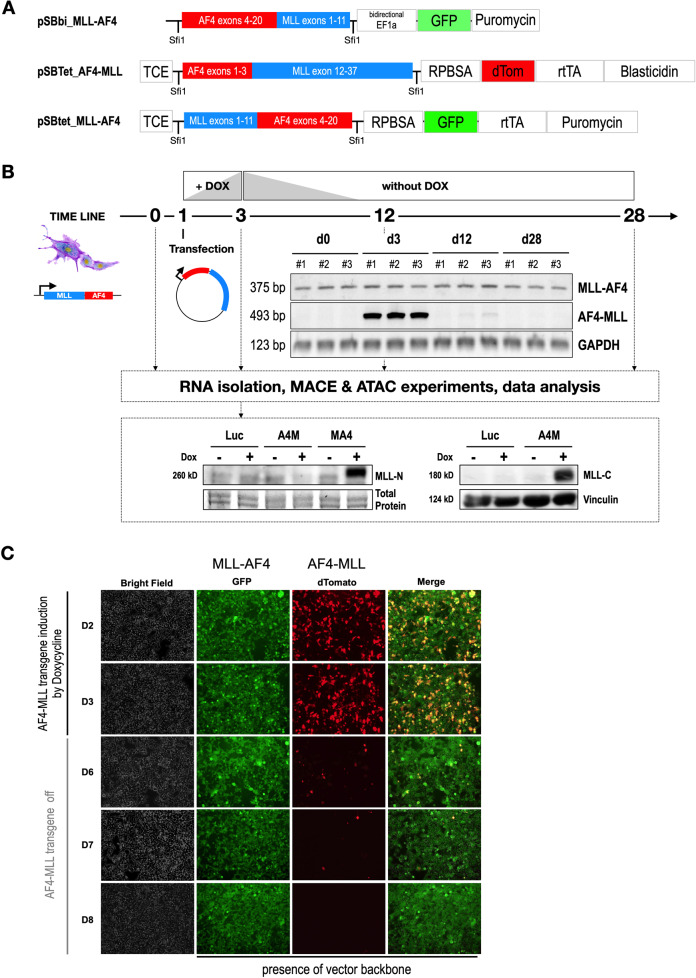
Fusions genes and established cell culture model. **A** All 3 vector constructs are depicted. Only the expressed part of the different Sleeping Beauty vectors is shown. The MLL-AF4 open reading frame was cloned into the constitutive pSBbi vector, while the reciprocal AF4-MLL expression construct was inserted into the pSBTet vector backbone. A final vector construct was the MLL-AF4 open reading frame inserted into the pSBTet-GP backbone; the latter construct was used for a control experiment during the ATAC-Seq experiment. **B** The construction of the test cell line is shown. HEK293 cells that constitutively express MLL-AF4 were used to transiently transfect the AF4-MLL construct at day 1. After a short selection and induction of the AF4-MLL transgene, the expression of AF4-MLL was shut down at day 3, and cells were grown for an additional 25 days. Samples for RNA or DNA isolation were taken at d0, d3, d12, and d28, respectively. RT-PCR experiments of all biological replicates are shown. While the MLL-AF4 fusion allele was expressed constantly over the observation period, the AF4-MLL fusion gene was diminishing. A GAPDH control demonstrates that equal amounts of RNA were used throughout this experiment. Western blot experiments were performed on day 3 for MLL-AF4 and AF4-MLL respectively. **C** Microscopic pictures to demonstrate the presence of all vectors as outlined. The SB vector encoding MLL-AF4 expresses constitutively GFP, while the inducible AF4-MLL vector expresses a dTomato protein. This way, the out-segregation of the AF4-MLL plasmid could be visually traced until day 7.

The t(4;11) fusion gene cassettes were cloned into existing Sleeping Beauty vector systems [21]. This resulted in 3 different constructs depicted in Fig. 1A: (1) pSBbi::*MLL-AF4* (*MLL* exons 1–10::*AF4* exons 4–20), (2) pSBTet::*AF4-MLL* (*AF4* exon 1–3::*MLL* exons 12–37) and (3) pSBTet::*MLL-AF4* (*MLL* exons 1–10::*AF4* exons 4–20). In order to develop a cell line model system that allowed us to address the above-mentioned scientific questions, we stably transfected the pSBbi::MLL-AF4 vector into HEK293T cells (ATCC CRL-3216™). These cells were then used to isolate the first RNA samples (d0) and were then transiently transfected with pSBTet::AF4-MLL (d1). Doxycycline-induction (1 µg/ml for 48 h) was carried out to achieve a population of cells that expressed both fusion genes. The expression of the reciprocal AF4-MLL fusion protein was terminated on day 3 by a medium exchange without Doxycycline. Cells were grown for an additional 25 days and aliquots were taken on days 3, 12, and 28, respectively. All this is summarized in Fig. 1B, where the RT-PCR experiments are shown for all experimental timepoints (d0 - d28; all three biological replicates). Strong expression of the *AF4-MLL* transgene was visible on day 3, before the shutdown of expression of this transgene. This was independently shown by the decreasing amount of red-fluorescent cells from d2 to d7 (see Fig. 1C). A Western blot experiment performed on day 3 demonstrated the co-expression of both MLL-AF4 and AF4-MLL, respectively (Fig. 1B). Isolated total RNA from d0–d28 in biological replicates was used to perform the MACE-Seq experiments.

### MACE analyses revealed again the synergism between the MLL-AF4 and AF4-MLL fusion proteins

**Fig. 2:**
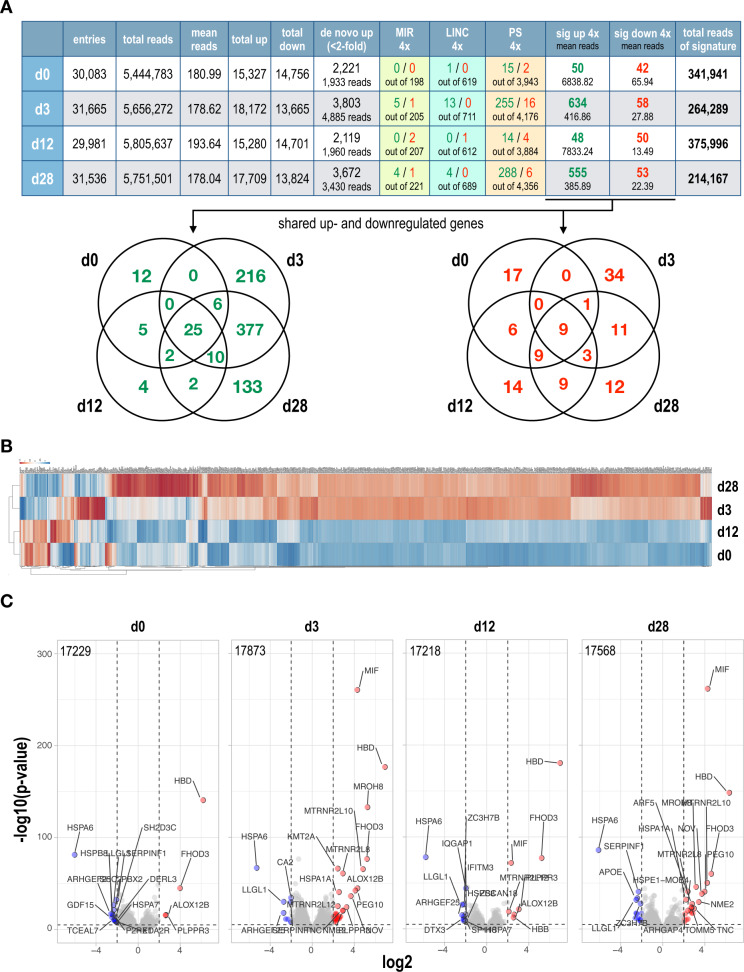
Data dissection of the RNA-Seq data and heatmap and volcano plot analysis. **A** Upper panel: summary of the MACE-Seq data. The first 6 columns summarize the data obtained by MACE-Seq, the last 6 columns display the filtered information, when applying stringent criteria (>10 reads, *p*-value < 0.05 log2 > ± 2) Bottom panel: VENN diagrams displaying the shared up- and downregulated genes between the different signatures. **B** Heatmaps were created by using the gene signatures (~700 up-and downregulated genes from all 4 timepoints) obtained from the cell lines at the four independent time points (d0, d3, d12, and d28) using the ClustVis online tool (biit.cs.ut.ee/clustvis/). **C** Similarly, gene entries of all protein-coding genes at the four independent time points (d0, d3, d12, and d28) were used to visualize the significant changes by volcano plots. Gene symbols together with *p*-values, log2 changes, and −log10 (*p*-value) data were used to perform the analyses (VolcaNoseR website, huygens.science.uva.nl). The number of gene entries used for the displayed plots is displayed in the upper left corner. We used stringent parameters to visualize in red and in blue the most significant changes in gene expression.

The overall MACE data analysis is summarized in Fig. 2A (upper panel). It summarizes the identified number of gene entries for all 6 cell lines. The last 6 rows display the significant signatures that were identified (>10 reads, *p*-value < 0.05 and FC > ±4).

The constitutive expression of the MLL-AF4 fusion protein alone caused a tiny signature of 50 upregulated and 42 downregulated genes. Co-expression of MLL-AF4 together with the reciprocal fusion protein AF4-MLL resulted in a highly increased gene set (634 upregulated, 58 downregulated genes). After turning off the AF4-MLL fusion protein, this large signature disappeared again on day 12 (48 upregulated, 50 downregulated genes) but re-appeared on day 28 (555 upregulated, 53 downregulated genes). This looked like a selection process where a subpopulation of cells from day 3 was positively selected overtime to maintain the extended day 3 gene signature.

Further inspection of these signatures (last row in Figs. 2A and S1, upper panel), revealed that the signature caused by MLL-AF4 resulted in 341,941 reads that derived from 15 pseudogenes (33.058 reads), 4 non-annotated genes (54 reads), 1 LincRNA gene (16 reads) and 13 protein-coding genes (3.083 reads). The vast majority of reads are derived from mitochondrial genes (*n* = 17; 305,728 reads). The downregulated gene signature was composed of 2 pseudogenes, 1 non-annotated gene, and 38 protein-coding genes with a total of 2769 reads.

The signature deriving from the co-expression of MLL-AF4 and AF4-MLL at day 3 exhibits a total of 264,289 reads. The majority of reads derived from upregulated pseudogenes (*n* = 255; 110,862 reads), non-annotated genes (*n* = 235; 45,116 reads), 13 LincRNA genes (328 reads), 5 microRNA genes (1831 reads), and 2 SnoRNA genes (23 reads). The protein-coding genes (*n* = 107) caused 22,656 reads, while mitochondrial genes were also highly expressed (*n* = 11; 83,435 reads). The downregulated gene signature was composed of 16 pseudogenes, 13 non-annotated genes, 1 microRNA gene, 2 SnoRNA genes, and 24 protein-coding genes with a total of 1616 reads.

The signature at day 12 was strongly reduced but still comprises 375,996 reads. The signature was derived from 14 pseudogenes (43.329 reads), 4 non-annotated genes (53 reads), and 11 protein-coding genes (9.328 reads). The vast majority of reads derived again from mitochondrial genes (*n* = 19; 323,283 reads). The downregulated gene signature was composed of 4 pseudogenes, 6 non-annotated genes, 1 LincRNA gene, 2 microRNA genes, and 36 protein-coding genes with a total of 674 reads.

The signature at day 28 expanded again and was composed of 214,167 reads. The signature derived from 288 pseudogenes (93.989 reads), 186 non-annotated genes (31,028 reads), 4 LincRNA genes (80 reads), 4 microRNA genes (1497 reads), 3 SnoRNA genes (51 reads), and 51 protein-coding genes (14.799 reads). Still, the vast majority of reads derived again from mitochondrial genes (*n* = 17; 72,853 reads). The downregulated gene signature was composed of 6 pseudogenes, 7 non-annotated genes, 1 microRNA gene, and 37 protein-coding genes with a total of 1186 reads.

From this type of analysis, we conclude that the presence of the AF4-MLL fusion protein enabled transcription of genes that are usually shut-down, e.g., pseudogenes, non-annotated genes, etc. This type of arbitrary gene activation can be clearly seen on day 3 and day 28. The Venn diagrams displayed in Fig. 2A (lower panels) show the overlap between signatures at day 3 and 28 (418 genes), while the downregulated gene sets appear to be idiosyncratic at each tested timepoint.

### Heatmap and volcano plot analyses revealed the high similarity between the day3 and day 28 signatures

For heatmap analyses, we retrieved only the protein-coding genes of all signatures. The heatmap analysis is displayed in Fig. 2B, where we analyzed deregulated target genes from all 4 timepoints. The combined gene set contained a total of 608 genes that were retrieved from the up- and down-regulated gene signatures at all timepoints. From the heatmap analysis, it became clear that cells expressing both fusion proteins at day 3 were clustering together on day 28, while the day 0 signature clustered together with day 12.

Similarly, we performed Volcano plot analyses with all protein-coding genes sets that are summarized in Fig. 2C. The total number of gene entries is indicated for each plot. Of interest, *MLL/KMT2A* is only visible at day 3, because of the overexpression of *AF4-MLL* that exhibits the poly-adenylated 3′-portion of the *MLL* gene. Another interesting finding is the *MIF* gene that can only be found to be strongly and significantly overexpressed in cells from d3–d28 (day 0 FC = 4.0, day 3 FC = 21.5, day 12 FC = 5.3, and day 28 FC = 21.3). High *MIF* expression (Macrophage Inhibitory Factor) has been recently linked to worse outcomes and high relapse in leukemia patients (see discussion).

### Involvement of mitochondrial gene activation by t(4;11) fusion proteins

One of the interesting findings was that certain mitochondrial genes were dramatically overexpressed, a mechanism that has never been described before for t(4;11) leukemia cells. This is summarized in Fig. S2, where mitochondrial genes are listed. This figure displays the number of reads from mock cells, as well as from the gene signatures obtained at d0–d28, respectively. The strong increase of distinct mitochondrial genes appears to be a property of the MLL-AF4 fusion protein, but could even be enhanced by the presence of the AF4-MLL fusion protein (e.g., ribosomal RNA genes). The most affected mitochondrial genes were *ATP6*, *CO1-3*, *CYB*, *ND4*, and both mitochondrial rRNAs (12S and 16S), respectively. Our experimental efforts to measure differences in mitochondrial respiration in our day 0 to day 28 cells versus mock cells remained inconclusive (data not shown). Therefore, we concluded that the overexpression of these mitochondrial genes has a yet unknown, metabolic function that occurs in the presence of t(4;11) fusion proteins.

### Chromosome usage analysis revealed patterns revealing the pathomolecular power of the different t(4;11) fusion genes

Another analysis we performed was to fingerprint the deregulated genes on chromosomes (GUDC module). This is depicted in Fig. S3, where the analysis is shown for all 4 timepoints for the observed up- and down-regulated gene signatures. The different timepoints are displayed and the “mean gene usage” is given (e.g., the signature of 50 up-regulated genes at day 0 has a mean of 0,11% of all genes from all chromosomes at that timepoint). Deviations from the mean usage are given for each chromosome (except chromosome Y). Relatively more target genes were expressed e.g., from chromosomes 18, 5, 11, and 21 at day 0.

The co-expression of AF4-MLL increased the mean gene usage from 0.11% to 1.96% (~18-fold increase), and some chromosomes showed a higher target gene number (chromosomes 5, 7, 2, 18, 1, 6), others with lower target gene numbers, (chromosomes 19, 17, 14, 20, 13, 12, 15, 8). Thus, target gene activation or repression is not a random but rather a selective process. Gene usage dropped again at d12, and the pattern at day 28 appears to be a further development of the day 3 pattern, indicating a clonal evolution that is presumably taking place.

### Comparison of MACE and ATAC-Seq data revealed a critical function of the reciprocal fusion protein AF4-MLL

Finally, we performed ATAC-Seq experiments to investigate the chromatin changes mediated by the expression of the single and co-expressed t(4;11) fusion proteins. For the purpose of our studies, we performed two different ATAC-Seq experiments. For the first experiment, we used an analogous setting to the MACE experiment. In the second experiment, we also analyzed both single fusion proteins (MLL-AF4 or AF4-MLL) in inducible vectors to address their functions in a setting where they were expressed individually. The transgenes in these single fusion protein experiments were induced for only 48 h and followed until day 28 to understand their individual impact.

**Fig. 3:**
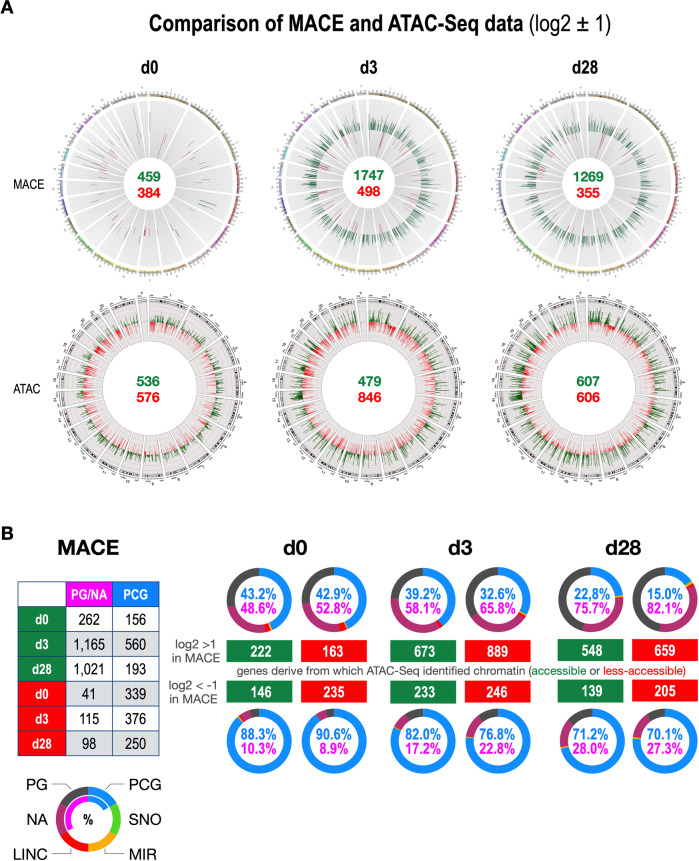
Comparing MACE- and ATAC-Seq data of the t(4;11) model system in Circos plots and DAGT module analysis. **A** Comparison of the genome-wide MACE- with ATAC-Seq data visualized for day 0, day 3, and day 28. It shows the number of deregulated genes in MACE experiments, as well as the changes in chromatin accessibility in the ATAC-Seq experiments. **B**. Left Table: summary of deregulated gene classes (pseudogenes/non-annotated genes (PG/NA)) versus protein-coding genes (PCG) for up and downregulated genes with a log2 values of ±1. Right part: Pseudogene/non-annotated genes (pink numbers) were compared to PCG’s (blue numbers) by indicating their percentages in each circle plot. Numbers in the green and red rectangles show e.g., that the up- or downregulated genes and their origin from accessible or less accessible chromatin fragments. This type of analysis was performed for all 12 subsections.

The resulting chromatin data from the first ATAC-Seq experiment (Fig. S1, lower panel) were quite comparable (see mean reads/gene entry). All data entries were then filtered to select target-gene signatures (>2 reads, *p*-value < 0.05, log2 > ± 1 or ± 2) and displayed by Circos plots in Fig. 3A, where significant signatures of MACE- and ATAC-Seq data are compared (log2 = ± 1). In MLL-AF4 expressing cells, the total number of deregulated genes was lower than the ATAC-Seq changes. Cells co-expressing both fusion proteins displayed already a much higher number of deregulated target genes when compared with the ATAC-Seq data. Similarly, the number of deregulated genes at d28 was again much higher than the observed changes in the ATAC-Seq experiment. This argues again that AF4-MLL acts like a “chromatin opener” as visible from the increasing chromatin accessibility from d3 to d28.

In order to investigate this assumption in more detail, we carefully analyzed the MACE- and ATAC-Seq data sets (Fig. 3B). MACE-Seq signatures were subclassified according to gene types (pseudogenes, non-annotated genes, LincRNA genes, microRNA genes, SNO genes, and protein-coding genes). In particular, the number of pseudogenes/non-annotated genes (PG/NA) and protein-coding genes (PCG) were summarized for the up- and downregulated gene signatures in the small table on the left of Fig. 3B. Noteworthy, not every target gene identified in the MACE experiment was also found in the ATAC-Seq experiment. However, the concordance was quite high (>90%). At day 0, target genes (385 out of 459 MACE targets) displayed a pattern that most activated genes derived from the accessible chromatin fractions, and vice versa, downregulated target genes (381 out of 384 MACE targets) were associated with less accessible chromatin. This changed at day 3, because activated target genes (1592 out of 1747 MACE targets) were more deriving from less accessible chromatin, while down-regulated genes (479 out of 498 MACE targets) could be attributed equally to both chromatin fractions. At day 28, the ratio for up-regulated genes (1207 out of 1269 MACE targets) was similar to d3, and downregulated genes (344 out of 355 MACE target genes) were again associated with less accessible chromatin. This clearly indicated that AF4-MLL allowed activating target genes even from the less accessible chromatin fraction.

**Fig. 4:**
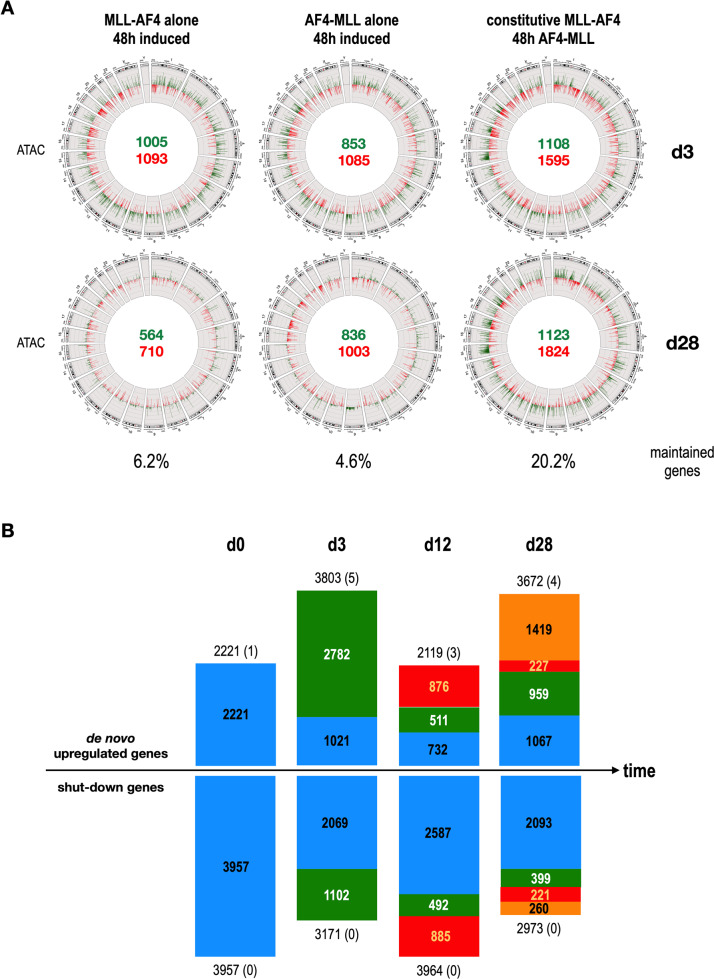
Changes in ATAC-Seq experiment with single fusion proteins and co-expression of both fusion proteins. **A** Circos plots of ATAC-Seq experiments with inducible MLL-AF4, inducible AF4-MLL, or the combination of constitutive MLL-AF4 in combination with inducible AF4-MLL reveal that the combination of constitutive MLL-AF4 and 48 h AF4-MLL demonstrated again the long-term impact on chromatin of this particular combination. Inducible transgenes were expressed only for 48 h and analyzed on day 3 and day 28 (inducible transgenes shut down for 25 days). **B** Analysis of de novo genes and shut-down genes by the DAGE/ST module. Several thousand genes were found to be induced or completely repressed by the expression of the tested t(4;11) fusion proteins. These signatures obtained at the individual timepoints were traced during the 4 weeks of the experiment and displayed by different colors (day 0 signature = blue; day 3 signature = green; day 12 signature = red; day 28 signature is orange). These analyses revealed a clonal evolutionary process that could very likely have been triggered by the expression of AF4-MLL on days 1–3.

In the second ATAC-Seq experiment, MLL-AF4 or AF4-MLL were only short-time induced and then turned off again (Fig. 4A). This was compared to our initial setting with a constitutive MLL-AF4 and 48 h expression of AF4-MLL. Short-term effects on chromatin at d3 were observed for both single-transfected cells, however, this effect was nearly lost on d28. When compared to cells in which both fusion proteins were present (constitutive MLL-AF4, inducible AF4-MLL for 48 h). the changes in chromatin accessibility were still detectable at d28. The maintained gene set at d28 was significantly higher than within the single transfected cells (20.2% versus 6.2% or 4.6%). This supported a “hit & run mechanism” exerted by AF4-MLL because the observed changes did not diminish after shutting down the expression of the AF4-MLL fusion protein. Importantly, these data suggest again the fact that an initial co-expression of both fusion proteins has a benefit, however, the AF4-MLL fusion could be lost once the process of clonal evolution has been initiated.

### De novo gene activation or the shut-down of gene transcription reveal an important mechanism of MLL fusion proteins

Finally, we investigated the obtained MACE-Seq data for de novo gene expression, as well as for the shut-down of genes in the four different signatures (DAGE & ST module). As shown in Fig. 4B, several thousand genes became either activated (2221 de novo genes at d0; 3803 at d3; 2119 at d12; 3672 at d28) or shut down (3957 shut-down genes at d0; 3171 at d3; 3964 at d12; 2973 at d28) in the presence of an individual or both fusion proteins at the four different time points. Except for a few genes (numbers in brackets), these de novo or shut-down gene signatures were not part of the highly up- or down-regulated gene signatures, as they are expressed at very low levels (with very few reads per gene). Moreover, these signatures could trace them over time. Vice versa, we observed similar behavior with genes that were completely turned off. Genes found to be shut down at day 0 could be traced until day 28, but again at days 3, 12, and 28 additional genes were shut down over time. This type of analysis was quite important as it contradicted the results presented in Fig. 2A–C. All these figures have suggested that the d28 signature derived from the day 3 signature, and was probably positively selected over 25 days (see above). However, the DAGE/ST module analyses of de novo and shut-down genes tell another story, namely that of ongoing evolution and selection that started at the timepoint when the reciprocal fusion protein AF4-MLL was expressed. This is a completely different view and is not apparent when analyzing heatmaps, volcano plots, or gene signatures. This ongoing evolution occurs at the level of lowly expressed genes and suggests that cells initiated such an evolutionary process as a result of the presence of the AF4-MLL fusion protein.

Therefore, we decided to examine these signatures in more detail. The upregulated protein-coding genes were analyzed in a VENN diagram (Fig. S4A). The overlap of these protein-coding genes was 95 genes in all four signatures. All subsignatures (overlapping and idiosyncratic) were analyzed by gene ontology enrichment analysis, but only those which are marked in green resulted in successful output. These were the gene sets with 41, 95, 541, 189, and 315 de novo genes, as well as the full signatures at day 3 (1195) and day 28 (889). Of interest, gene set 541 contained an “innate response program” (response to bacteria; antimicrobial response) as well as a “humoral immune response”, while the full signature of de novo genes at day 28 displayed “B cell proliferation”. In addition, the major pathways identified on days 3 and 28 were “G-protein-coupled receptor signaling”, “Regulation of signaling receptor activity” and “Calcium signaling”, respectively. This is interesting, as it points to unexpected signaling pathways. As these signatures were mostly related to the presence of AF4-MLL, we can assume that the changes made by this fusion protein are probably important.

To verify these initial findings, we also examined the protein-coding genes in the traceable signatures shown in Fig. S4B. Again, we extracted the protein-coding genes and analyzed these gene sets by pathway analyses. The blue signature did not reveal any known pathway. The green signature at day 3 revealed the pathways already identified, “G-protein-coupled signaling”, “innate cell response” and “humoral immune response”. Of interest, only the combination of two protein-coding gene signatures (orange and green) at day 28 revealed the “B cell proliferation” pathway. In addition, “Calcium homeostasis”, “Chloride transport” and “PLC-activating G-protein signaling” pathways became overt. All this indicates that the cells were changing their behavior and diverging significantly from the original cells at day 0 due to clonal evolution.

We did the same analysis for the shut-down genes to determine which programs were switched off. In Fig. S5A we first analyzed the shut-down gene signatures in a VENN diagram and analyzed all intersections and idiosyncratic signatures by pathway analyses. Most of these analyses for intersections and full signatures revealed that “cell adhesion”, “cell migration” or “cell-cell interactions” are lost from these cells. The idiosyncratic program at day 3 (153 genes) also revealed a relief of “cell fate commitment”, which by contrast would suggest a “cell de-differentiation process”.

Further analysis of the traceable genetic shut-down program is summarized in Fig. S5B. Here, it became obvious that the “biological” or “cell adhesion” as well as “migration” were already shut down at d0. The signature at d3 also inhibited cell-cell interactions, while the MLL-AF4 mediated signature at d12 was focussed on inhibiting “T cell functions”. Combining these signatures at d3 and d12 supports this finding (blue/green at d3 and d12). The green signature at d28 once more inhibits “T helper cell functions”.

In summary, the analysis of de novo and shut-down genes over the four different time points clearly suggests a clonal evolution of these cells when triggered by the expression of the reciprocal fusion protein AF4-MLL for 48 h (d1–d3). The induced changes in the genetic program trigger the cells towards B cell pathways and inhibit T cell functions, and moreover inhibit migration and cell adhesion processes, while supporting PLC- and G-protein coupled signaling pathways, which are important for several chemokine signaling pathways.

In conclusion, the expression of AF4-MLL between d1 and d3 induced a gene signature of which a large portion (~80%) was still present at d28. Of interest, most identified genes that were upregulated at d3 or d28 represent pseudogenes or non-annotated genes, indicative of non-specific activation of chromatin which may allow other transcription factors, e.g., the MLL-AF4 fusion protein, to activate novel gene signatures that may promote the conversion of normal cells into pre-malignant cells. To this end, AF4-MLL prepares the ground for an adaptive cell type that may change according to internal or external signals.

## Discussion

This manuscript describes experiments addressing two important questions concerning the pathology of t(4;11) MLL fusion proteins. First of all, we aimed to understand the pathological relevance of the direct and reciprocal fusion protein, MLL-AF4 and AF4-MLL, on a genome-wide level. The second question addressed whether continued expression of AF4-MLL is required, or whether AF4-MLL could act by a hit-and-run mechanism. The latter question was raised by several observations, namely that some patients with t(4;11) leukemia are diagnosed with an *MLL-AF4* fusion gene, but lack the *AF4*-*MLL* allele. Several laboratories have already shown that the missing *AF4*-*MLL* allele could be explained by complex translocations [22], but there are also cases where the reciprocal fusion gene is not expressed (either transcriptionally inactive or deleted allele). A recent publication has also demonstrated that t(4;11) patients lacking the expression of the *AF4*-*MLL* allele have a worse prognosis [20].

To address both questions at the same time, we decided to use a setting where MLL-AF4 is constitutively expressed, while AF4-MLL expression could be temporarily turned on by using an inducible vector backbone.

Constitutive expressing MLL-AF4 alone resulted in a relatively small signature (for details see Fig. 2A). When AF4-MLL was co-expressed for a very short time period (48 h), an immense upregulation of target genes occurred. The observed gene signature contained roughly 8-times more protein-coding genes, but also several hundred pseudogenes and non-annotated genes. Such a strong increase of genes could only be explained by non-specific chromatin activation that enabled this massive increase of gene transcription. The heatmap analysis (Fig. 2B) clearly clustered d0 with d12, and d3 with d28, while the volcano plot analysis (Fig. 2C) already indicated certain genes to be expressed from day 3 onward, indicating already here that the expression of AF4-MLL initiated an ongoing clonal evolution (e.g., the disappearance of ALOXB12 at day 12, or the maintenance of MIF (d3–d28)). MIF has been recently identified as a critical target gene that correlated with a worse outcome in leukemia patients, as it was defined as an independent prognostic factor important for OS and DSF [23].

A detailed analysis of the ATAC-Seq data is displayed in Fig. S1 (lower panel). At day 0, most gene types (PS, NA, LINC, MIR, SNO, and PCG) were equally up- and down-regulated. From d3 onwards, the number of all gene types that were now increasingly linked with accessible chromatin (PG, NA, LINC, and PCG). This indicated again that the transcriptional patterns were evolving and become selected over time, due to short-term expression of the AF4-MLL fusion protein (d1–d3).

The detailed comparison of MACE- and ATAC-Seq data (Fig. 3A/B) revealed a clear shift from protein-coding genes to pseudogenes/non-annotated genes in the upregulated signatures. This could well be interpreted as an evolutionary process where many genes were activated for gene transcription, even from less accessible chromatin. It seems that genes are tested for any benefit they may offer to these cells, with genes not supporting a cellular advantage presumably being shut down. That these genes are derived from the pool of pseudogenes or non-annotated genes is not a surprise since those genes may harbor benefits for malignant cell growth [24]. Thus, it seems that the cells are gambling with their genetic coding potential in order to find the best way to adapt to a new cell fate. In biology, this is equivalent to “cell plasticity” and is usually a typical sign of stem or progenitor cells.

This evolutionary process can not be maintained by the single fusion proteins alone, as seen in Fig. 4A. Thus, co-expression of both fusion proteins– even when giving them only a short time of action—seems to be a necessary step in order to induce changes in the chromatin that remain over longer time periods.

This interpretation is supported by findings within the low expressed gene signatures (de novo and shut-down genes by using the DAGE/ST module; Fig. 4B). We could clearly see in the identified de novo and shut-down gene signatures that the expression of AF4-MLL induced an evolutionary process. Of note, we observed within the de novo gene fractions a “B-cell specific” gene signature, while genes responsible for “T-cell activities” were downregulated at d28 (Figs. S4 and S5). In addition, from day 3 onwards, we saw gene signatures that resemble innate immune cell activities, and thus, recalls a “myeloid gene program”. This would fit perfectly the well-known mixed-lineage phenotype of t(4;11) leukemia cells.

As a side note, strong overexpression of specific mitochondrial genes (ATP6, CO1-3, CYB, ND4, and both mitochondrial rRNAs (12S and 16S)) were overexpressed in the presence of t(4;11) fusion proteins (Fig. S2). We have no rational explanation for this experimental observation, despite the fact that others have observed that the overexpression of certain mitochondrial genes was associated with poor clinical outcomes [25, 26].

Based on these data, we pose the hypothesis that the disruption of the MLL protein between the CXXC domain and the PHD/BD domain causes a dramatic effect: it results in a direct fusion protein that is able to strongly enhance target gene transcription, but that the additional presence of a complementary, reciprocal fusion protein AF4-MLL enables a “broader use” of the genome, namely the activation of certain genes within repressed chromatin. Such an “adaptive genome usage” would be important, as it allows a given cell to change its cell fate rapidly, depending on triggers from the outside. These novel features make a pre-tumor cell almost omnipotent with regard to deregulated gene expression. Over time and depending on external signals, this will convert a normal cell into an aberrant cell, and most likely causes the onset of cancer, combined with strong features of pluripotency. According to our experience with *MLL-r* leukemias, this is presumably one of the best definitions we can make for the most prevalent *MLL-r* leukemias, namely those bearing a t(4;11) translocation.

## Material and methods

### Cell culture and transfections

HEK293T cells were grown in DMEM with 10% (v/v) FCS (Capricon Scientific), 2 mM L-Glutamine (Capricon Scientific), and 1% (v/v) Pen Strep (GE Healthcare) at 37 °C and 5% CO_2._ The single-transfected stable cell line expressing *MLL-AF4* (pSBbi::MLL-AF4) in a constitutive fashion was established by using a low amount (50 ng) of SB transposase vector SB100X. After 24 h, cells were subjected to Puromycin (1 µg/ml). The cells were incubated with selection markers for 3–10 days and terminated when virtually all cells were emitting the expected green color derived from their corresponding reporter genes (eGFP). The cells were further cultivated for several weeks before being used for the second round of transient transfection with an inducible *AF4-MLL* construct (pSBTet::AF4-MLL). Blasticidin and Doxycycline were administered only for 48 h and then relived to allow segregation. The transfected cell line continued to express the respective reporter and selection marker (eGFP and Puromycin), while the second plasmid became out-segregated due to lack of selection.

### RNA extraction, cDNA synthesis, and RT-PCR experiments

The cell line expresses the *MLL-AF4* transgene throughout the 4-weeks observation time. The *AF4-MLL* transgene was induced by using 1 µg/ml Doxycycline to the cell culture for 48 h. Total RNA was isolated using RNeasy^®^ Mini Kit (Qiagen) and cDNA synthesis was performed using SuperScript^®^ II (Invitrogen) at days 0, day 3, day 12, and day28. All isolated RNAs were quality checked (Agilent Bioanalyzer) and final concentrations were determined. Equal amounts of total RNA were used throughout all experiments, and all experiments were performed with 3 biological replicates. All primers used for RT-PCR analyses are as follows: MLL8•3 (5′–CCCAAAACCACTCCTAGTGAG–3′), MLL13•5 (5′–CAGGGTGATAGCTGTTTCGG–3′), AF4•3 (5′–GTTGCAATGCAGCAGAAGCC–3′), and AF4•5 (5′–ACTGTCACTGTCCTCACTGTCA–3′). With these 4 oligonucleotides, transcription of all vector-derived transgenes was successfully tested.

### Differential gene expression profiling by MACE-Seq

The chimeric genes were either expressed constitutively or induced for 48 h with 1 µg/ml Doxycycline and total RNA were isolated from transfected cell lines. After testing the correct expression of both transgenes, differential gene expression (DGE) profiles were obtained by MACE (Massive Analysis of cDNA Ends)—Seq experiments following the manufacturer protocol (GenXPro, Frankfurt, Germany). Further details are given in the Supplementary data file. The MACE data have been deposited on the GEO server with the Accession number GSE178569.

### ATAC-Seq experiments

Preparation of ATAC samples was performed according to a published protocol [27]. Further details are given in the Supplementary data file. The ATAC-Seq data have been deposited on the GEO server with the Accession number GSE178567 and GSE178568.

### Outline of our experimental setting and bioinformatic pipeline: data evaluation and establishment of novel tools

As summarized in Fig. S6, our experimental setting was used to perform MACE- and ATAC-Seq experiments. Differential expression analysis was performed using the R-Bioconductor DESeq2 library. Raw counts were normalized by the geometric mean-based method [28]. These data were used to define a simple algorithm (>10 reads, *p*-values < 0.05, log2 > ± 2) that allows the definition of highly significant gene signatures. The resulting data were used to prepare Circos plots [29] for the visualization of genome-wide changes in gene transcription or the ATAC-Seq data. In addition, we used these data sets to generate heatmaps, volcano plots, and pathway analyses.

In addition, we used the FileMaker database program to import all the DESeq2 data for further analysis and to apply additional algorithms. This resulted in three additional analytic modules, named GUDC, DAGT, and DAGE/ST, respectively. The GUDC module analyzes the “gene usage on different chromosomes”, which results in a kind of “chromosome fingerprint”. The result of the analysis is displayed for each chromosome as more (positive) or less (negative) gene expression in comparison to the mathematical mean expression for each chromosome. The DAGT module (“differential analysis by gene type”) automatically subclassifies each gene entry in our signatures to one of the different gene types (pseudogenes, non-annotated genes, LINC RNAs, MIR RNAs SNO RNAs, mitochondrial genes, and protein-coding genes. Finally, the DAGE/ST module “differential analysis of de novo or shut-down gene expression”) uses the DESeq2 data to identify “de novo induced genes” or “shut-down genes” after t(4;11) transgene expression. For this purpose, a log2_var_ discriminator (defined as “Ln(fold change)/Ln2”) was used, because the DESeq2 provides log2 data even when mock or experimental data displayed zero reads. By using the log2_var_ discriminator, we were able to quickly identify all “de novo transcribed genes” or “shut-down genes” and included these critical gene sets in our analyses. The “signature tracing” (ST) module allows tracing gene signatures over time.

## Supplementary information

Supplementary data

MACE-Seq day 0

MACE-Seq day 3

MACE-Seq day 12

MACE-Seq day 28

ATAC-Seq day 0

ATAC-Seq day 3

ATAC-Seq day 28

Comparison ATAC-Seq say 3 with day 28
