# Supplementary material for: The role of reciprocal fusions in MLL-r acute leukemia: studying the chromosomal translocation t(4;11)
Source: Oncogene. 2021 Sep 6;40(42):6093–102. doi: 10.1038/s41388-021-02001-2 (PMC8530991; doi:10.1038/s41388-021-02001-2)
Supplement: Supplementary file 1 — Supplementary data [file 41388_2021_2001_MOESM1_ESM.pdf]

Supplemental Data file

Supplemental data files (Excel):

The first set of 4 files (MACE-data) contains the gene entries with gene identifier, gene symbol, gene name, mean exp, mean mock, fold change log2, pvalue, -log10(pvalue), var-log2, chromosome number (with beginning and end), classification (into pseudogene (PG), non-annotated gene (NA), LINC RNA gene (LINC), MIR gene (MIR), SNO gene (SNO), MT gene (MT) and protein coding gene (PCG)). All data are given as in the Bioconductor output file with the exception of var-log2 which was simply calculated by ln(mean exp/mean mock)/ln2. The following files are provided:

1. Day 0 gene signature.xlsx
2. Day 3 gene signature.xlsx
3. Day 12 gene signature.xlsx
4. Day 28 gene signature.xlsx

The second set of 3 files (ATAQ-data) contain the gene entries with gene identifier, gene symbol, gene name, mean exp, mean mock, fold change log2, pvalue, var-log2, chromosome number (with beginning and end), classification (into pseudogene (PG), non-annotated gene (NA), LINC RNA gene (LINC), MIR gene (MIR), SNO gene (SNO) and protein coding gene (PCG)). All data are given as in the Bioconductor output file with the exception of var-log2 which was simply calculated by ln(mean exp/mean mock)/ln2. The following files are provided:

5. ATAC Day 0.xlsx
6. ATAC Day 3.xlsx
7. ATAC Day 28.xlsx
8. all-comparison_d3 vs d28_ATAQ

Methods

ATAC-Seq experiments

In particular, cells were grown in 6-well plate and treated with 1 µg/ml Doxycyclin for 48 hours. After that cells were harvested, and viability were checked in every samples. In all samples viability were more than 90%. Cells were then resuspended in cold PBS and cell numbers in each sample were counted. 50,000 cells were centrifuged at 500 RCF for 5 minutes at 4°C in a fixed angle centrifuge. After centrifugation, 900 µl of supernatant was aspirated with P1000 pipette and the remaining 100 µl of supernatant was carefully aspirated by pipetting with a P200 pipette tip to carefully avoid the cell pellet. Cells were resuspended in 50 µl cold ATAC-Seq Resuspension Buffer (RSB; 10 mM Tris-HCl pH 7.4, 10 mM NaCl, and 3 mM $MgCl_2$ in water) containing 0.1% NP40, 0.1% Tween-20, and 0.01% Digitonin, pipetting up and down 3 times. Cells were incubated on ice for 3 minutes and then 1 ml of cold ATAC-Seq Resuspension Buffer (RSB) containing 0.1% Tween-20 but no NP-40 and Digitonin was added. Tubes were inverted 3 times to mix and then centrifuged at 500 RCF for 10 minutes at 4°C in a fixed angle centrifuge to pellet down the nuclei. Supernatant was removed with two pipetting steps, as described before, and nuclei were resuspended in 50 µl of transposition mix (25 µl 2× TD buffer (20 mM Tris-HCl pH 7.4, 10 mM $MgCl_2$, 20 % Dimethyl Formamide in water), 2.5 µl transposase 26 (100 nM final), 16.5 µl PBS, 0.5 µl 1% digitonin, 0.5 µl 10% Tween-20, and 5 µl water) by pipetting up and down six times. Transposition reactions were incubated at 37°C for 30 min in a thermomixer with shaking at 1000 rpm. The final ATAC-Seq experiment was performed at GenXPro (https://genxpro.net/) that used an Illumina HiSeq for the analysis, and the resulting data were analysed by bioinformatics tools.

Differential gene expression profiling by MACE-Seq and Bioinformatic analyses

The chimeric genes were either expressed constitutively, or induced for 48h with 1 µg/ml Doxycycline and total RNA were isolated from transfected cell lines. After testing the correct expression of both transgenes, differential gene expression (DGE) profiles were obtained by MACE (Massive Analysis of cDNA Ends) - Seq experiments following the manufacturer protocol (GenXPro, Frankfurt, Germany). Resulting data from 3 biological replicates of the cell line at the 4 different time points (day 0, day 3, day 12 and day 28) were compared with 3 biological replicates of mock-transfected cells. The MACE-libraries were prepared at GenXPro GmbH using the Massive Analysis of cDNA Ends (MACE) Library Preparation Kit (v2.0) from GenXPro GmbH. First, cDNA was generated using Oligo(dT) primers with distinct Oligo IDs per sample for subsequent pooling of up to 24 samples. After pooling, cDNA was fragmentated to an average size of 200 bp using the sonicator Biorupter Plus (Diagenode, Belgium). The distribution of cDNA fragment sizes was monitored using the automated microfluidic electrophoresis station LabChip GXII Touch HT platform (PerkinElmer, USA). The poly(A) containing cDNA fragments were purified using solid phase reversible immobilization (SPRI) beads (Agencourt AMPure XP, USA), end repaired and ligated to distinct 8-base pair UMI Adapters (also called TrueQuant adapters). Then, the library containing labelled and fragmentated cDNA was amplified by PCR, purified by SPRI beads (Agencourt AMPure XP, USA) and strand-specific sequenced using the HiSeq2500 (Illumina, USA).

Bioinformatic analysis was performed according to the analysis pipeline for MACE libraries by GenXPro GmbH. Unique Oligo IDs and UMIs on each transcript allowed initial demultiplexing and subsequent removal of PCR-duplicates. The remaining reads were trimmed for high-quality as well as adapter-free sequences and aligned to the human reference genome (Genome Reference Consortium Human Build 38 patch release 13 (GRCh38.p13) using Bowtie 2. Resulting output data were implemented in the database program FileMaker for further analysis. All data received from the Bioconductor software from our RNA-Seq or ATAC-Seq experiments were incorporated into a FILEMAKER database program. All final data sets were exported from the FILEMAKER Database program as individual Excel documents for publication. Moreover, thr FILEMAKER Database program was used to develop the GUDC, DAGT and DAGE/ST modules. In addition, we used the following server for further data analysis: ClustVis (https://biit.cs.ut.ee/clustvis/) for heatmap analyses; VolcaNoseR (https://huygens.science.uva.nl/VolcaNoseR/) for volcano plots; ShinyGO v0.61 (http://bioinformatics.sdstate.edu/go/) for pathway analyses.

Supplemental Figure legends:

Figure S1: Data dissection of the MACE- and ATAC-Seq experiment

The dissected data obtained from our MACE- and ATAC-Seq experiments are summarized. Upper panels display the identified gene signatures from days 0 - 28; the gene signatures were dissected into pseudogenes (PG), non-annotated genes (NA), LincRNA genes (LINC), microRNA genes (MIR), SNO genes (SNO), mitochondrial genes (MT) and protein coding genes (PCG), respectively. Lower panels display the dissected ATAC-Seq data. In the first panel, total number of entries, and the number of accessible (total up) and non-accessible chromatin (total down) is displayed. The last 4 columns represent the total number of the up- and down-regulated chromatin regions and their associated genes. These were calculated for up- and downregulated chromatin regions by a minimum of 2 reads, a p-value < 0,05 combined with log2 changes of >2 in case of up-regulated genes, while downregulated genes were identified by a minimum of 2 reads in the mock sample, a p-value < 0,05 combined with a log2 value of < -2. In the lower panel, the same ATAC-Seq data was dissected for the number of pseudogenes (PG), non-annotated genes (NA), LincRNA genes (LINC), microRNA genes (MIR), SNO genes (SNO), mitochondrial genes (MT) and protein coding genes (PCG), respectively. Green and red numbers represent the total number of identified and classified up- and down-regulated signatures, respectively.

Figure S2: Profiling mitochondrial gene transcription

Several mitochondrial genes were strongly transcribed in cells that express MLL-AF4 or both t(4;11) fusion proteins, even when the AF4-MLL transgene was shut down after day 3. Among those genes, ATP6, ATP8, CO1-CO3, CYB, ND4, as well both ribosomal RNAs RNR1 and RNR2 were highly expressed. It is not clear what kind of physiological consequences this causes in the cells, because respiration experiments did not show any significant difference between mock cells and cells expressing these t(4;11) fusion proteins.

Figure S3: GUDC module analysis

All 4 gene signatures were analyzed for the number of genes that were deregulated on each chromosome and displayed as percentages of all genes present on the same chromosome. **Left:** up-regulated genes in the t(4;11) cell culture model at all 4 timepoints (day 0 = blue; day 3 = green; day 12 = red; day 28 = orange). **Right:** down-regulated genes. For each timepoint the “mean of deregulated genes for all chromosomes” is given in percent. The graphs display a ‘fingerprint’ of the gene usage on each chromosome. The resulting graphical pattern can be used to deduce the genome-wide impact of the tested fusion proteins. It also demonstrates that target gene usage is not a random process.

Figure S4: Venn diagram and detailed pathway analysis of de novo genes

A. *De novo* genes signatures were analyzed along all 4 timepoints. All idiosyncratic and shared signature genes were analyzed by pathway analysis. Those where pathways could be identified are indicated by green numbers, while for all grey numbers, no pathway was identified. Pathways were found for days 3 and 28, as well intersections exhibiting 41, 95 and 189 genes, respectively. These pathways revealed G protein-coupled signaling, including Ca2+ signaling, as well as innate and humoral immune response, as well as B cell proliferation.

B. the Signature Tracing module revealed the same pathways, but could in addition attribute these pathways to the protein coding genes that arose at d3 and the combined signatures that derive from d3 and d28.

Figure S5: Venn diagram and detailed pathway analysis of shut-down genes

Shut-down genes signatures were analyzed along all 4 timepoints. All idiosyncratic and shared

signature genes were analyzed by pathway analysis. Those where pathways coukld be identified are indicated by red numbers, while for all grey numbers, no pathway was identified. Pathways were found for days 0, 3, 12 and 28. These signatures all show 3 major pathways, namely adhesion, migration and secretion. Idisosyncratic and intersection signatures - exhibiting 202 and 153 genes - identified again innate immune pathways and cell adhesion.

B. the Signature Tracing module revealed the same pathways (adhesion and migration), but could in addition attribute these pathways to the protein coding genes that arose at d0 and d3, while downregulated T-cell specific functions could be attributed to the d0-derived signature at day 12, while T-helper immune functions derived from the d3-signature at day28.

Figure S6:

MACE or ATAC-Seq experiments were performed and resulting data were analyzed by Bioconductor software to create output Excel files. Various other bioinformatic tools were used to analyze these data (volcano plots with huygens.science.uva.nl; heatmaps with biit.cs.ut.ee/clustvis; pathway analsyis with bioinformatics.sdstate.edu). Circos plots display the genome-wide changes identified by RNA-Seq or ATAC Seq experiments. Finally, all Bioconductor output files were imported to the Filemaker database program, which allowed the creation of novel modules for refined analyses not provided by the Bioconductor package (GUDC, DAGT, DAGE/ST).

Figure S1

MACE data dissection

d0 upregulated genes (n=50)						
PG	NA	LINC	MIR	SNO	MT	PCG
15	4	1	0	0	17	13

d0 downregulated genes (n=42)						
PG	NA	LINC	MIR	SNO	MT	PCG
2	1	0	0	0	1	38

d3 upregulated genes (n=634)						
PG	NA	LINC	MIR	SNO	MT	PCG
255	52	13	5	2	14	92

d3 downregulated genes (n=58)						
PG	NA	LINC	MIR	SNO	MT	PCG
16	14	0	1	2	2	23

d28 upregulated genes (n=554)						
PG	NA	LINC	MIR	SNO	MT	PCG
287	204	4	4	3	16	36

d28 downregulated genes (n=53)						
PG	NA	LINC	MIR	SNO	MT	PCG
5	7	0	1	0	2	38

ATAC Seq data dissection

	total entries	total up	total down	log2 > 1	log2 > 2	log2 < -2	log2< -1
d0	46.127	23.092	23.035	536	243	281	576
d3	48.129	19.627	28.502	479	189	432	846
d28	53.103	24.357	28.746	607	314	180	606

	total entries	PG	NA genes	LINC	MIR	SNO	PCG
d0	46.127	3.718	7.151	921	417	105	10.786
		3.834	7.248	773	368	72	10.736
d3	48.129	2.632	5.763	696	226	77	10.236
		5.676	9.572	1.018	675	117	11.450
d28	53.103	3.949	7.819	955	250	80	11.308
		6.223	9.739	894	1.047	179	10.672

Figure S2

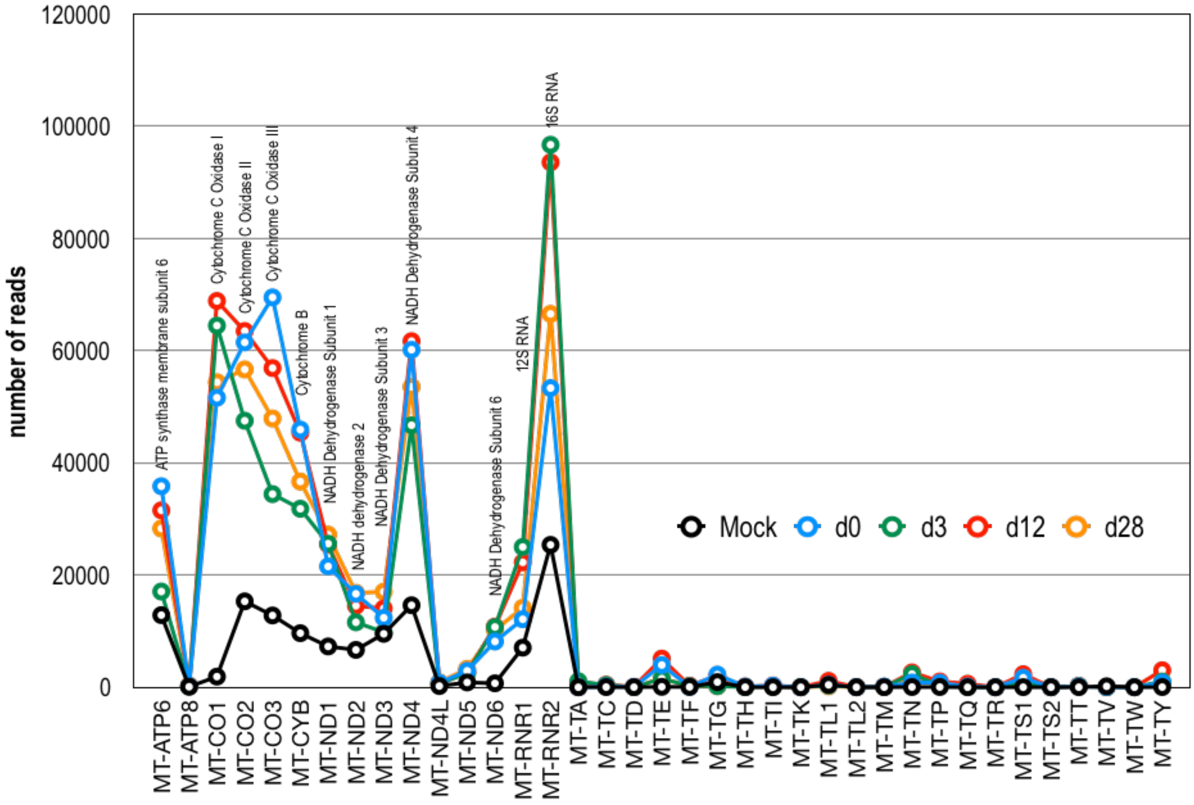

Figure S3

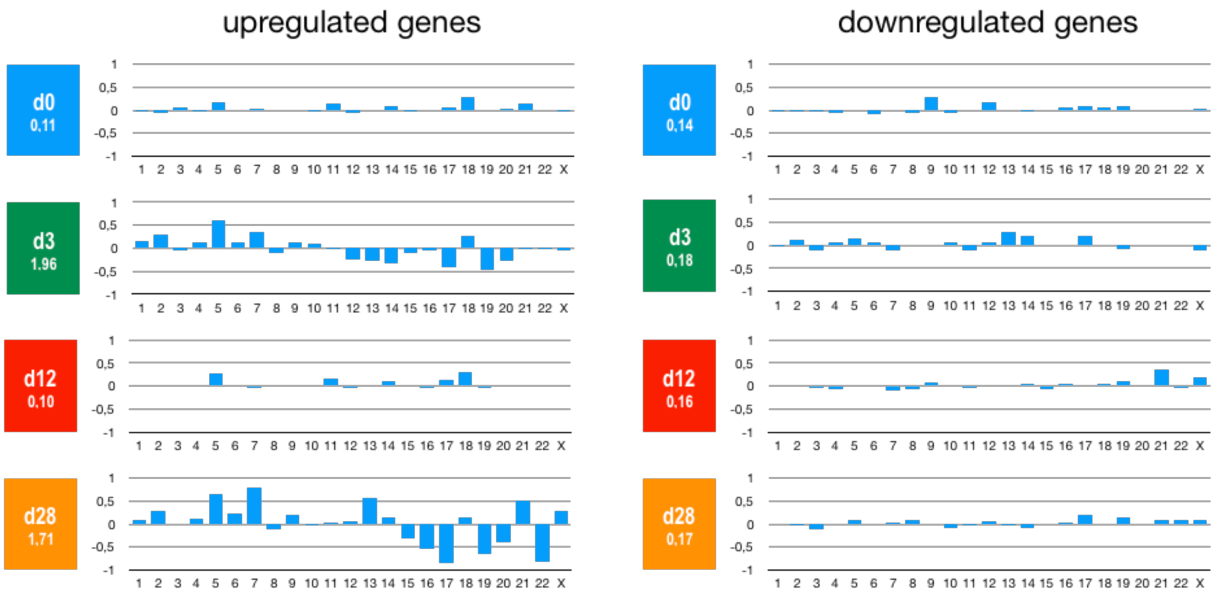

Figure S4

S4A

De novo genes

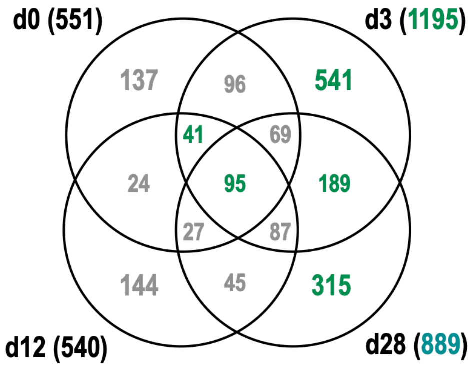

Enrichment FDR	# genes	functional category 1195
0.00731800278203	120	**G protein-coupled receptor signaling pathway**
0.01781093431012	63	**Response to bacterium**
0.02537759974096	59	Regulation of signaling receptor activity
0.00947586880911	54	Defense response to other organism
0.00177706237761	38	Humoral immune response

# genes	Functional Category 541
61	**G protein-coupled receptor signaling pathway**
34	**Response to bacterium**
19	**Humoral immune response**

# genes	Functional Category 41
2	Peptidoglycan catabolic process

# genes	Functional Category 95
4	Gas transport
3	Oxygen transport

# genes	Functional Category 189
4	DNA methylation involved in gamete generation

# genes	Functional Category 315
11	Inorganic anion transport

Enrichment FDR	# genes	functional category 889
0.008006710676578	50	**Regulation of signaling receptor activity**
0.031034211305113	37	Calcium ion homeostasis
0.008006710676578	32	Regulation of cytosolic calcium ion concentration
0.005378293263702	16	PLC-activating G protein-coupled receptor signaling
0.031034211305113	15	Inorganic anion transmembrane transport
0.038195915750487	13	**B cell proliferation**

S4B

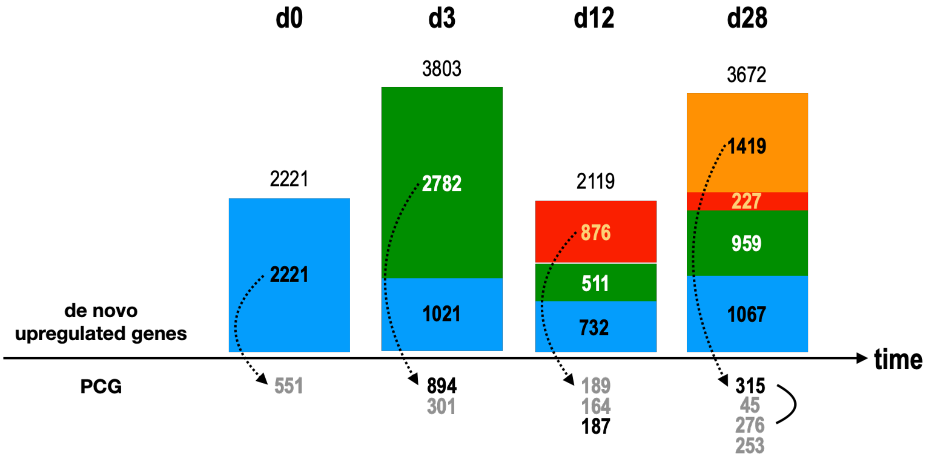

Enrichment FDR	# genes	functional category 894
0.004025406462861	96	**G protein-coupled receptor signaling pathway**
0.044072509934388	65	Multi-organism reproductive process
0.039367791547801	64	Response to other organism
0.039461568129720	64	Response to external biotic stimulus
0.014267977077045	50	**Response to bacterium**
0.013707834455872	48	Regulation of signaling receptor activity
0.014820831446505	42	Defense response to other organism
0.004025406462861	30	**Humoral immune response**
0.049684727863224	28	Regulation of cytosolic calcium ion concentration

Enrichment FDR	# genes	functional category 187
0.02314512539739	4	Gas transport

Enrichment FDR	# genes	functional category 315
0.02533017224534	11	Inorganic anion transport
0.02533017224534	9	Inorganic anion transmembrane transport
0.02533017224534	9	Chloride transport

Enrichment FDR	# genes	functional category 315/276
0.0474837305496395	64	**G protein-coupled receptor signaling pathway**
0.0216419609663946	37	Regulation of signaling receptor activity
0.0474837305496395	27	Calcium ion homeostasis
0.0216419609663946	24	Regulation of cytosolic calcium ion concentration
0.0216419609663946	12	PLC-activating G protein-coupled receptor signaling
0.0224028025241963	12	Chloride transport
0.0474837305496395	10	**B cell proliferation**

Figure S5

S5A

shut-down genes

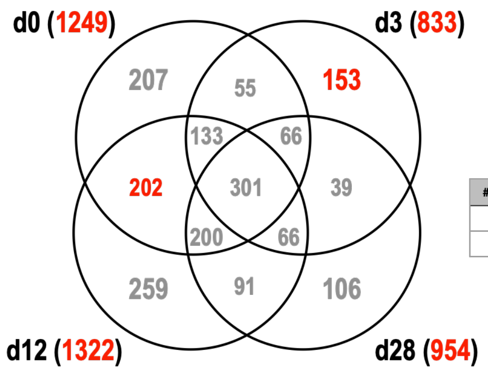

Enrichment FDR	# genes	functional category 1249
0.0485266467823053	118	Biological adhesion
0.0485266467823053	117	Cell adhesion
0.0122873623602966	65	Regulation of signaling receptor activity
0.0485266467823053	28	Positive regulation of ERK1 and ERK2
0.0485266467823053	19	Lymphocyte migration

Enrichment FDR	# genes	functional category 883
0.0121645371880987	94	Cell adhesion
0.0121645371880987	94	Biological adhesion
0.0274811711548385	47	Regulation of signaling receptor activity
0.0181771792299656	26	Cell-cell adhesion via plasma-membrane

# genes	Functional Category 153
10	Cell-cell adhesion via adhesion molecules
3	Negative regulation of cell fate commitment

# genes	Functional Category 202
10	Defense response to bacterium
4	Response to lipoprotein particle
4	Positive regulation of acute inflammatory response

Enrichment FDR	# genes	functional category 1322
0.00335898792450523	134	Biological adhesion
0.00335898792450523	133	Cell adhesion
0.0288579881492706	64	Regulation of signaling receptor activity
0.0444810446074521	49	Leukocyte migration
0.0461718553358326	46	Positive regulation of secretion by cell
0.0330323388826899	42	Extracellular matrix organization

Enrichment FDR	# genes	functional category 954
0.041404329183934	17	Acid secretion

S5B

Enrichment FDR	# genes	functional category 1249
0.04852664678230	117	Cell adhesion
0.01228736236029	65	Regulation of signaling receptor activity
0.04852664678230	28	Positive regulation of ERK1 and ERK2
0.04852664678230	19	Lymphocyte migration
0.04852664678230	7	Thymic T cell selection

Enrichment FDR	# genes	functional category 328
0.02943492192037	15	Cell-cell adhesion via adhesion molecules

Enrichment FDR	# genes	functional category 836
0.04122306062445	6	Thymic T cell selection
0.03870220620224	5	Negative thymic T cell selection
0.04057322068956	5	Positive thymic T cell selection

Enrichment FDR	# genes	functional category 328/555
0.0121645371880987	94	Cell adhesion
0.0274811711548385	47	Regulation of signaling receptor activity
0.0181771792299656	26	Cell-cell adhesion via plasma-membrane
0.0181771792299656	19	Homophilic cell adhesion via plasma membrane
0.0181771792299656	16	Acid secretion

Enrichment FDR	# genes	functional category 836/136
0.00660462736242602	101	Biological adhesion
0.00763438039496412	100	Cell adhesion
0.0490373279722105	49	Regulation of signaling receptor activity
0.0490373279722105	16	Lymphocyte migration

Enrichment FDR	# genes	functional category 350
0.02201563465982	12	Tissue remodeling

Enrichment FDR	# genes	functional category 105
0.02228278669509	4	T-helper 1 type immune response

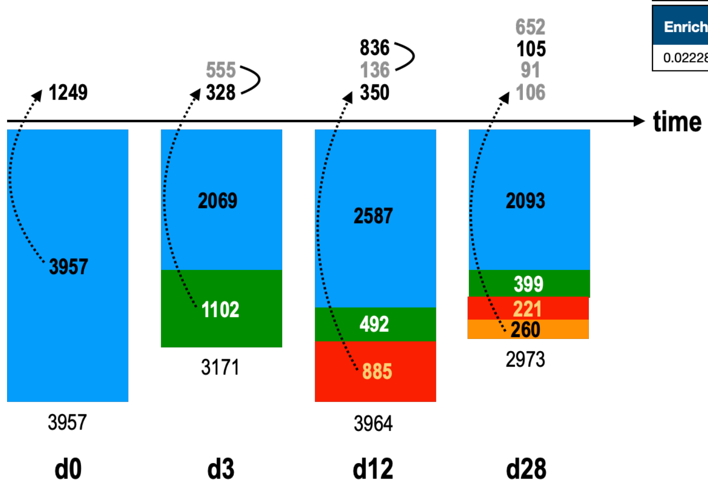

Figure S6

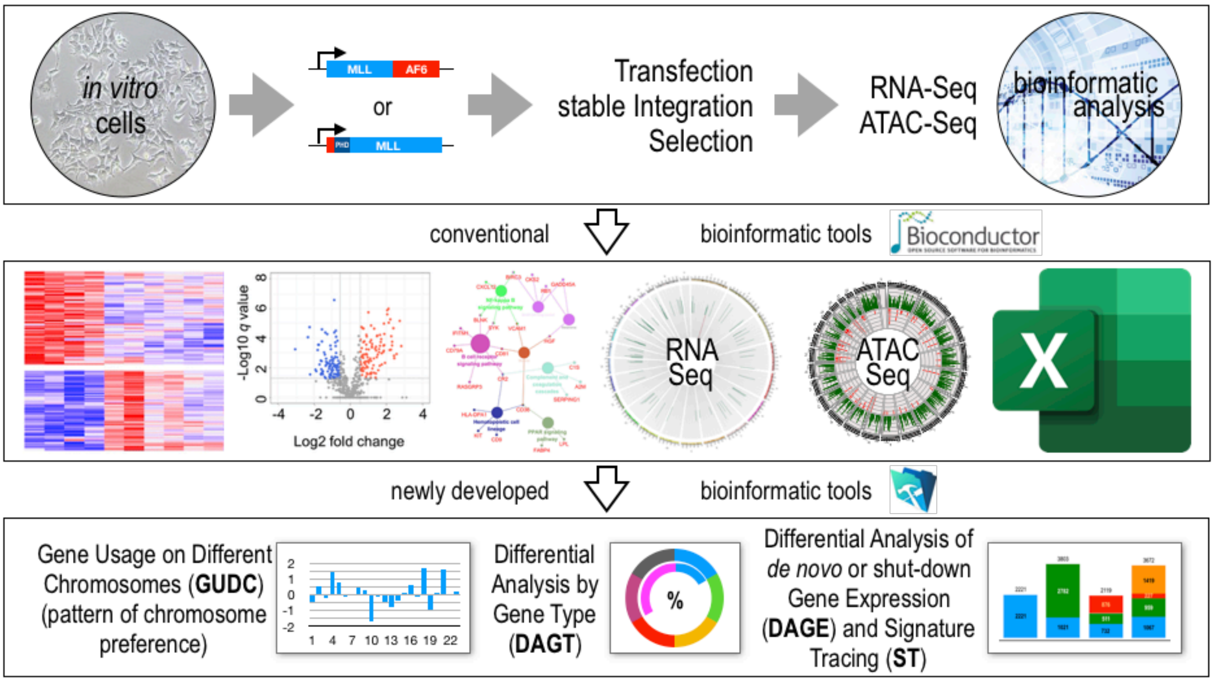
in vitro cells
MLL
AF6
or
PHD
MLL
Transfection stable Integration Selection
RNA-Seq ATAC-Seq
bioinformatic analysis
conventional
bioinformatic tools
Bioconductor
-Log10 q value
Log2 fold change
RNA Seq
ATAC Seq
newly developed
bioinformatic tools
Gene Usage on Different Chromosomes (GUDC) (pattern of chromosome preference)
Differential Analysis by Gene Type (DAGT)
%
Differential Analysis of de novo or shut-down Gene Expression (DAGE) and Signature Tracing (ST)
